# Draft Genome Sequence of Lignin-Degrading *Agrobacterium* sp. Strain S2, Isolated from a Decaying Oil Palm Empty Fruit Bunch

**DOI:** 10.1128/MRA.00259-21

**Published:** 2021-05-13

**Authors:** Ummu Habibah Faisal, Nurul Syazwani Ahmad Sabri, Nurtasbiyah Yusof, Analhuda Abdullah Tahir, Nuurul Nadrah Mohd Said, Fatimah Azizah Riyadi, Fazrena Nadia Md Akhir, Nor’azizi Othman, Hirofumi Hara

**Affiliations:** a Department of Chemical and Environmental Engineering, Malaysia-Japan International Institute of Technology, Universiti Teknologi Malaysia, Kuala Lumpur, Malaysia; b Department of Mechanical Precision Engineering, Malaysia-Japan International Institute of Technology, Universiti Teknologi Malaysia, Kuala Lumpur, Malaysia; University of Arizona

## Abstract

We report the draft genome sequence of *Agrobacterium* sp. strain S2, isolated from a decaying oil palm empty fruit bunch (OPEFB) in Negeri Sembilan, Malaysia, which yields potential genes encoding lignin degradation enzymes. This genome of 9,722,071 bp exhibited 58.9% GC content, 10,416 coding genes, and 12 RNAs.

## ANNOUNCEMENT

Structurally, lignin comprises amorphous, complex, and cross-linked polymers composed of phenylpropane monomers bonded with C-O and C-C linkages, providing biomass recalcitrance (1). Over the years, profound efforts have been made in the conversion of lignocellulose to second-generation biofuels and value-added chemicals (2, 3). These efforts have led to the continuous isolation of lignin-degrading bacteria from various habitats (2–8). Considering that this specific strain was isolated from a decaying oil palm empty fruit bunch (OPEFB) source, which is known to harbor a substantial amount of lignin (29.2%) compared to that in hardwood (15 to 24%), softwood (20 to 27%), and other agricultural waste (12 to 25%) (9), the genome of *Agrobacterium* sp. strain S2 could potentially elucidate the genes that allow for lignin degradation.

An OPEFB left for 6 months was collected from an oil palm plantation in Negeri Sembilan, Malaysia (2°45′48.6″N, 101°46′10.6″E). An aliquot of serially diluted stock solution containing 1.0 g of freshly cut decaying OPEFB and 0.7% NaCl was plated onto agar plates enriched with W minimal medium and 2.5 g/liter alkali lignin as the sole carbon source prior to incubation at 30°C for 7 days to obtain pure colonies (10). Genomic DNA of *Agrobacterium* sp. strain S2 was extracted using a Qiagen DNA minikit from a single colony grown overnight in 5 ml LB medium at 30°C and 160 rpm. A high-quality 400-bp library was first constructed via an Ion Xpress Plus fragment library kit and checked using an Agilent 2100 bioanalyzer system according to the manufacturer’s protocol. The diluted library was subjected to template preparation using the Ion Chef system. An Ion 530 chip kit was utilized for sequencing alongside the Ion S5 XL sequencing system. After 4 h of sequencing following the loading of enriched Ion Sphere particles onto the Ion 530 chip, raw data analysis, alignment, and variant calling were performed via Torrent Suite software. Sequencing yielded 3,024,751 reads with an average read length of 375 bp, and reads were then subjected to Qiagen CLC Genomics Workbench software version 11.0.1 for quality trimming and assembly. The parameters used were as follows: quality score limit, 0.05; maximum number of ambiguous nucleotides, 2; and discarded read, <400 nucleotides. Default parameters were used for the assembly.

The genome assembly yielded 3,253 contigs with a total size of 9,722,071 bp, an *N*_50_ value of 5,151 bp, and a GC content of 58.9%. Annotation of the genome was performed via the NCBI Prokaryotic Genome Annotation Pipeline (PGAP) (11) with 96,826 bp as the largest contig and a prediction of 10,416 coding genes, 12 RNAs, and 83 tRNAs (12). Further genome annotation administered via BLAST analysis against the UniProt (13) and Pfam 33.1 (13) databases revealed numerous genes encoding lignin-derived aromatic compound degradation enzymes, such as superoxide dismutase, cytochrome P450, catalase-peroxidase, nonheme chloroperoxidase, and benzaldehyde dehydrogenase. Aromatic ring-oxidizing genes such as 4-hydroxybenzoate polyprenyltransferase and 4-hydroxyphenylacetate 3-monooxygenase were also discovered. These findings indicate the lignin-degrading potential of *Agrobacterium* sp. strain S2 in the utilization and valorization of lignocellulose biomass and lignin waste.

### Data availability.

The complete genome sequence of *Agrobacterium* sp. strain S2 was deposited at GenBank under BioProject number PRJNA699083 and BioSample number SAMN17774993 with SRA accession number SRR13638391 and assembly number JAFEJZ000000000.

## References

[1] Santos RB, Hart P, Jameel H, Chang H-M. 2013. Wood based lignin reactions important to the biorefinery and pulp and paper industries. Bioresources 8:22. doi:10.15376/biores.8.1.1456-1477.

[2] Chang Y-C, Choi D, Takamizawa K, Kikuchi S. 2014. Isolation of Bacillus sp. strains capable of decomposing alkali lignin and their application in combination with lactic acid bacteria for enhancing cellulase performance. Bioresour Technol 152:429–436. doi:10.1016/j.biortech.2013.11.032. 24316485

[3] Priyadarshinee R, Kumar A, Mandal T, Dasguptamandal D. 2017. Exploring the diverse potentials of Planococcus sp. TRC1 for the deconstruction of recalcitrant kraft lignin. Int J Environ Sci Technol 14:1713–1728. doi:10.1007/s13762-017-1257-7.

[4] Wang D, Lin Y, Du W, Liang J, Ning Y. 2013. Optimization and characterization of lignosulfonate biodegradation process by a bacterial strain, Sphingobacterium sp. HY-H. Int Biodeterior Biodegradation 85:365–371. doi:10.1016/j.ibiod.2013.06.032.

[5] Xu R, Zhang K, Liu P, Han H, Zhao S, Kakade A, Khan A, Du D, Li X. 2018. Lignin depolymerization and utilization by bacteria. Bioresour Technol 269:557–566. doi:10.1016/j.biortech.2018.08.118. 30219494

[6] Goodner B, Hinkle G, Gattung S, Miller N, Blanchard M, Qurollo B, Goldman BS, Cao Y, Askenazi M, Halling C, Mullin L, Houmiel K, Gordon J, Vaudin M, Iartchouk O, Epp A, Liu F, Wollam C, Allinger M, Doughty D, Scott C, Lappas C, Markelz B, Flanagan C, Crowell C, Gurson J, Lomo C, Sear C, Strub G, Cielo C, Slater S. 2001. Genome sequence of the plant pathogen and biotechnology agent Agrobacterium tumefaciens C58. Science 294:2323. doi:10.1126/science.1066803. 11743194

[7] Rashid GMM, Durán-Peña MJ, Rahmanpour R, Sapsford D, Bugg TDH. 2017. Delignification and enhanced gas release from soil containing lignocellulose by treatment with bacterial lignin degraders. J Appl Microbiol 123:159–171. doi:10.1111/jam.13470. 28393443

[8] Si W, Wu Z, Wang L, Yang M, Zhao X. 2015. Enzymological characterization of Atm, the first laccase from Agrobacterium sp. S5-1, with the ability to enhance in vitro digestibility of maize straw. PLoS One 10:e0128204. doi:10.1371/journal.pone.0128204. 26010258 PMC4444218

[9] Isikgor FH, Becer CR. 2015. Lignocellulosic biomass: a sustainable platform for the production of bio-based chemicals and polymers. Polym Chem 6:4497–4559. doi:10.1039/C5PY00263J.

[10] Tahir AA, Mohd Barnoh NF, Yusof N, Mohd Said NN, Utsumi M, Yen AM, Hashim H, Mohd Noor MJM, Akhir FNM, Mohamad SE, Sugiura N, Othman N, Zakaria Z, Hara H. 2019. Microbial diversity in decaying oil palm empty fruit bunches (OPEFB) and isolation of lignin-degrading bacteria from a tropical environment. Microbes Environ 34:161–168. doi:10.1264/jsme2.ME18117. 31019143 PMC6594733

[11] Tatusova T, DiCuccio M, Badretdin A, Chetvernin V, Nawrocki EP, Zaslavsky L, Lomsadze A, Pruitt KD, Borodovsky M, Ostell J. 2016. NCBI Prokaryotic Genome Annotation Pipeline. Nucleic Acids Res 44:6614–6624. doi:10.1093/nar/gkw569. 27342282 PMC5001611

[12] Aziz RK, Bartels D, Best AA, DeJongh M, Disz T, Edwards RA, Formsma K, Gerdes S, Glass EM, Kubal M, Meyer F, Olsen GJ, Olson R, Osterman AL, Overbeek RA, McNeil LK, Paarmann D, Paczian T, Parrello B, Pusch GD, Reich C, Stevens R, Vassieva O, Vonstein V, Wilke A, Zagnitko O. 2008. The RAST Server: Rapid Annotations using Subsystems Technology. BMC Genomics 9:75. doi:10.1186/1471-2164-9-75. 18261238 PMC2265698

[13] Mistry J, Chuguransky S, Williams L, Qureshi M, Salazar Gustavo A, Sonnhammer ELL, Tosatto SCE, Paladin L, Raj S, Richardson LJ, Finn RD, Bateman A. 2020. Pfam: the protein families database in 2021. Nucleic Acids Res 49:D412–D419. doi:10.1093/nar/gkaa913. PMC7779014 33125078

